# Photocatalytic Hydrogen Generation in Surfactant‐Free, Aqueous Organic Nanoparticle Dispersions

**DOI:** 10.1002/smll.202406236

**Published:** 2024-10-02

**Authors:** Jan Bruder, Karen Fischer, Jonas Armleder, Erich Müller, Nicola Da Roit, Silke Behrens, Yuman Peng, Wolfgang Wenzel, Holger Röhm, Alexander Colsmann

**Affiliations:** ^1^ Karlsruhe Institute of Technology (KIT) Material Research Center for Energy Systems Strasse am Forum 7 76131 Karlsruhe Germany; ^2^ Karlsruhe Institute of Technology (KIT) Light Technology Institute Engesserstrasse 13 76131 Karlsruhe Germany; ^3^ Karlsruhe Institute of Technology (KIT) Institute of Nanotechnology Hermann‐von‐Helmholtz‐Platz 1 76344 Eggenstein‐Leopoldshafen Germany; ^4^ Karlsruhe Institute of Technology (KIT) Laboratory for Electron Microscopy Engesserstrasse 7 76131 Karlsruhe Germany; ^5^ Karlsruhe Institute of Technology (KIT) Institute of Catalysis Research and Technology Hermann‐von‐Helmholtz‐Platz 1 76344 Eggenstein‐Leopoldshafen Germany

**Keywords:** aqueous dispersions, hydrogen generation, organic nanoparticle dispersions, photocatalysis, surfactant‐free

## Abstract

Hydrogen generation in electrostatically stabilized, aqueous organic nanoparticle dispersions is investigated. For this purpose, organic nanoparticle dispersions are synthesized in water by nanoprecipitation from tetrahydrofuran and stabilized by charging through strong molecular electron acceptors. The dispersions are stable for more than 10 weeks on the shelf and during the photocatalytic process, despite the continuous transfer of charges between the reactants. The hydrogen generation in the electrostatically stabilized dispersions outperforms the hydrogen generation in organic nanoparticle dispersions which contain the common stabilizer sodium dodecyl sulfate.

## Graphical abstract

Photocatalytic hydrogen generation from aqueous organic semiconductor nanoparticle dispersions is demonstrated. Strong electron acceptors are employed to charge the nanoparticles, avoiding the use of surfactants. The dispersions are long‐term stable on the shelf and during operation.

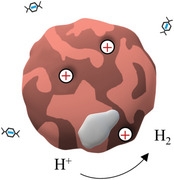

## 1 Introduction

Photocatalysis enables the direct production of hydrogen by irradiation of semiconductors with sunlight. Among the existing materials and device concepts, nanoparticle dispersions of photocatalysts potentially enable the lowest costs for hydrogen generation, because they exhibit the largest photocatalytic surface per volume of light‐harvesting material.^[^ ^1^ ^]^ Commonly, nanoparticulate ceramics are used for photocatalytic overall water splitting, such as TiO_2_ or carbon nitrides. Yet, their large bandgaps limit the use of solar energy to only a fraction of the solar spectrum. To mitigate these large bandgaps, the so‐called Z‐scheme is used, which involves two separate semiconductors with smaller bandgaps, one for the oxygen evolution reaction and one for the hydrogen evolution reaction. For the hydrogen evolution reaction, organic bulk‐heterojunction (BHJ) nanoparticles, comprising electron donors (e.g., linear conjugated polymers) and electron acceptors (e.g., fullerenes or so‐called non‐fullerene acceptors) to warrant efficient charge carrier separation, recently have made considerable progress.^[^ ^2^, ^3^ ^]^ The hydrogen generation in organic nanoparticle dispersions benefits from the tailored absorption spectrum and the high absorption coefficients of organic semiconductors.

So far, aqueous organic nanoparticle dispersions for photocatalytic hydrogen generation have been synthesized along the miniemulsion route. A disadvantage of this method is the tendency to produce nanoparticles with core‐shell structure, that is a donor‐rich core surrounded by an acceptor‐rich shell or vice versa, which can potentially limit the photocatalytic performance by trapping positive or negative charge carriers in the center of the nanoparticle.^[^ ^2^, ^4^ ^]^ To some extent, control over the internal morphology of the nanoparticles can be achieved by tailoring the surface energy of the nanoparticles.^[^ ^2^, ^5^ ^]^ An alternative approach to nanoparticle synthesis was reported related to the eco‐friendly solar cell fabrication from semiconductor dispersions in alcohols: within microseconds, nanoprecipitation often produces nanoparticles with well‐intermixed donor and acceptor phases.^[^ ^6^, ^7^ ^]^ For this purpose, the organic semiconductors are dissolved in a “good solvent” and then injected into a larger quantity of a miscible “poor solvent” (non‐solvent). The rapid reduction in semiconductor solubility then triggers the immediate formation of nanoparticles due to the oversaturation of the solvent mixture.

Since nanoparticles in dispersion tend to agglomerate and sediment, stabilization mechanisms must be employed. The synthesis along the miniemulsion route intrinsically requires the use of surfactants which later help to sterically stabilize the nanoparticles. Surfactants at the nanoparticle surface, however, may potentially reduce the photocatalytically active area and thus arguably limit the ultimate efficiency of the photocatalysis.^[^ ^8^ ^]^ In previous reports on organic solar cells fabricated from nanoparticle dispersions in alcohols, strong electron acceptors were used for electrostatic stabilization by charge transfer and electrostatic repulsion, hence avoiding the use of surfactants.^[^ ^9^ ^]^ If this concept can be translated to photocatalysis, the photocatalytically active surface can be maximized. The most obvious challenge when employing this concept of electrostatic stabilization is the maintenance of the dispersion stability under continuous charge carrier exchange with the environment during the photocatalytic process.

In this work, we present a study of photocatalytic hydrogen generation in surfactant‐free, electrostatically stabilized organic BHJ nanoparticle dispersions. This includes the synthesis of tailored electrostatically stabilized dispersions in water, which so far was only known in ethanol, methanol or acetonitrile.

## 2 Results and Discussion

### 2.1 Stabilization of P3HT Nanoparticles in Water

**Figure 1:**
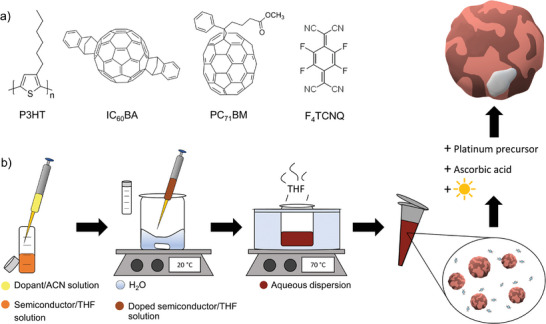
a) Chemical structures of P3HT, IC_60_BA, PC_71_BM, and F_4_TCNQ. b) Synthesis of electrostatically stabilized nanoparticle dispersions for hydrogen generation. Upon injection of the semiconductor solution (THF) into water under stirring, the semiconductor solubility is reduced instantly, and nanoparticles form. Then THF is thermally evaporated, leaving behind an aqueous dispersion. The co‐catalyst platinum is obtained by photodeposition onto the nanoparticle surfaces through the conversion of a platinum precursor in the presence of a sacrificial reagent (ascorbic acid).

For our study, we have deliberately chosen a BHJ comprising the light‐harvesting donor polymer poly(3‐hexylthiophene) (P3HT) and fullerene acceptors, all of which are depicted in **Figure** **1**a. While we acknowledge that more efficient light‐harvesting polymers are available, P3HT is best understood and can be supplied at competitive prices. The energy levels of BHJs from P3HT and fullerenes are suitable for hydrogen generation in the presence of a sacrificial reagent. Moreover, P3HT uniquely features the formation of intrinsically stable nanoparticle dispersions in alcohols due to self‐charging,^[^ ^10^ ^]^ and its charging can be further enhanced with a wide range of strong electron acceptors (i.e., dopants), such as 2,3,5,6‐tetrafluoro‐7,7,8,8‐tetracyanoquinodimethane (F_4_TCNQ).^[^ ^9^ ^]^

All dispersions were synthesized by nanoprecipitation omitting any stabilizing surfactants as illustrated in Figure 1b. For this purpose, P3HT was dissolved in tetrahydrofuran (THF, 1 mL, *c*_P3HT_ = 1 g L^−1^) and then rapidly injected into deionized water (3 mL) to trigger the formation of P3HT nanoparticles. After nanoprecipitation, the THF was evaporated from the dispersion in a water bath on a hotplate (70 °C) and the remaining water volume was further reduced to the primary volume of 1 mL. Prior to nanoprecipitation, an aliquot of F_4_TCNQ solution in acetonitrile (ACN) was added to the P3HT/THF solution. ACN was used to dissolve F_4_TCNQ to avoid side reactions with the solvent and maintain its neutral state.^[^ ^11^ ^]^ We note that our organic nanoparticle dispersions must be synthesized in water since the synthesis in a different agent followed by drying of the soft nanoparticles would irreversibly lead to the strong formation of agglomerates which prevent redispersing.

**Figure 2:**
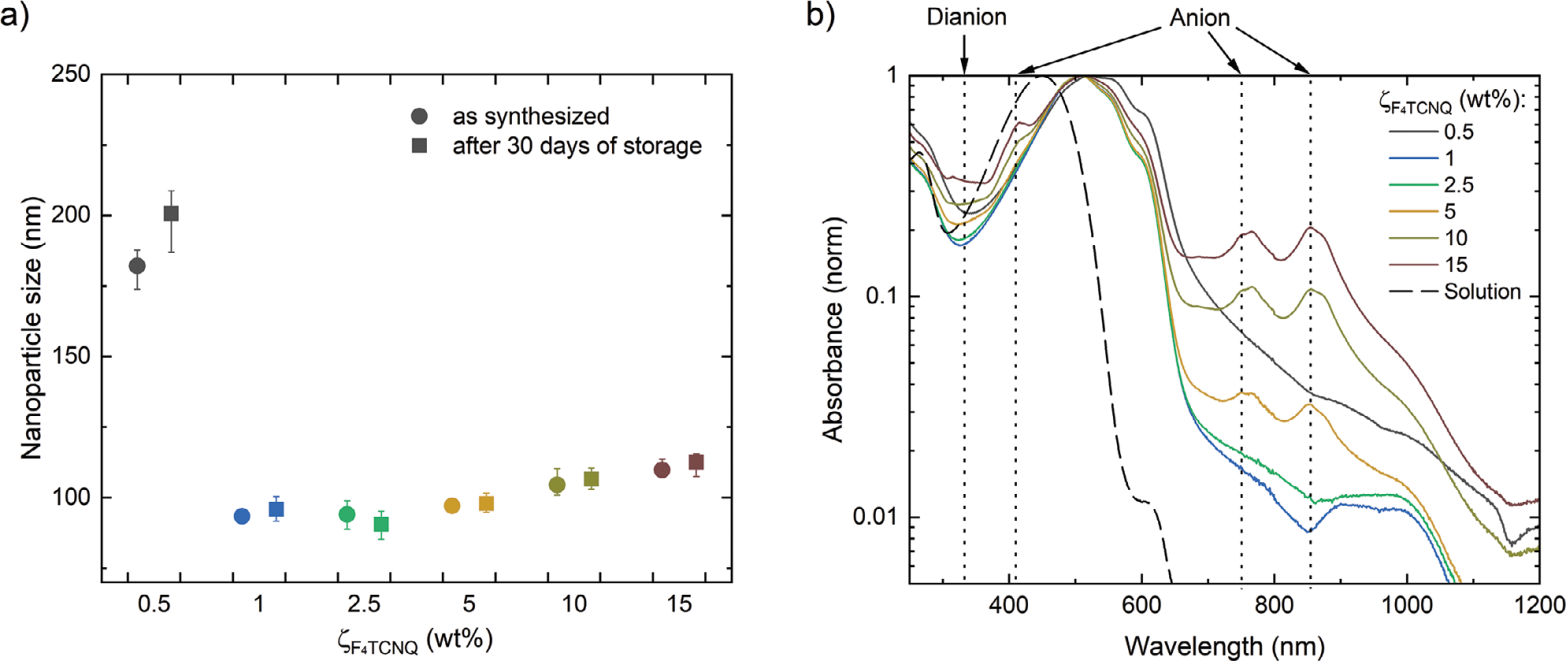
a) P3HT nanoparticle sizes in dependence of the F_4_TCNQ concentration *ζ*_F4TCNQ_. Sizes were determined immediately after synthesis of the dispersion and again after 30 days of storage in the dark. The error bars represent the measurement data range (min/max) of 12 repeating measurements on the same dispersion. b) Absorbance spectra of the dispersions on a logarithmic scale normalized to the P3HT peak and, for reference, the spectrum of a P3HT solution (chloroform, *c*_P3HT_ = 0.01 g L^−1^).

At the outset of our study, we optimized the concentration of F_4_TCNQ for best nanoparticle formation and electrostatic stability. At this stage, we used neat P3HT and omitted the fullerene, since the electrostatic stabilization by F_4_TCNQ only acts on P3HT, whereas the fullerene is passively nested inside the polymer.^[^ ^12^ ^]^ **Figure** **2**a (closed circles) depicts the size of the nanoparticles in dispersion (Z‐average, measured by dynamic light scattering, DLS) versus the concentration of F_4_TCNQ which we varied between *ζ*_F4TCNQ_ = 0 and *ζ*_F4TCNQ_ = 15 wt% (in relation to the mass of P3HT). If no F_4_TCNQ was added (*ζ*_F4TCNQ_ = 0 wt%), we did not obtain stable nanoparticle dispersions after the evaporation process, but P3HT agglomerated and formed large swimming patches that remained in the beaker (Figures S1a and S2a, Supporting Information). This observation is different from previous reports on the nanoprecipitation of P3HT from chloroform into ethanol or methanol where neat P3HT formed stable nanoparticle dispersions due to self‐charging.^[^ ^10^, ^13^ ^]^ Here, in water, F_4_TCNQ is required to extrinsically stabilize the dispersion. Notably, the nanoparticles formed in water were larger (*d* > 90 nm) than their counterparts that were previously nanoprecipitated from chloroform solution into ethanol (*d* < 70 nm).^[^ ^9^ ^]^ According to the scientific literature, THF and water mix slower than chloroform and ethanol.^[^ ^14^ ^]^ The slower mixing results in the formation of fewer polymer nuclei, which leads to fewer but larger nanoparticles. We note that some visible strip‐like agglomerates formed during volume reduction of the dispersion which floated on the water and later remained in the beaker, yet without affecting the stability of the dispersion at large (Figures S1b–d and S2b–d, Supporting Information).

We found the smallest nanoparticles (*d* = 94 nm) upon addition of F_4_TCNQ at *ζ*_F4TCNQ_ = 1 wt% and *ζ*_F4TCNQ_ = 2.5 wt%. This is consistent with the results of Manger et al., who achieved a nanoparticle size minimum at *ζ*_F4TCNQ_ = 2 wt% in ethanol.^[^ ^9^ ^]^ Smaller nanoparticles indicate more efficient charging and hence better stabilization of the dispersions as a larger total surface is produced.^[^ ^10^ ^]^ Toward smaller amounts of F_4_TCNQ (*ζ*_F4TCNQ_ = 0.5 wt%), the dispersions were stable, but the nanoparticle size was significantly larger (*d* = 180 nm) since fewer charges were transferred onto P3HT. However, if F_4_TCNQ is used in quantities exceeding *ζ*_F4TCNQ_ = 5 wt%, we also observed a minor increase of the nanoparticle size, despite the enhanced charging of P3HT. This observation is consistent with earlier works^[^ ^9^, ^10^, ^15^ ^]^ and probably stems from the formation of aggregates of doped P3HT.^[^ ^16^ ^]^

Once synthesized, all dispersions exhibited very good long‐term stability. As depicted in Figure 2a (closed squares), the nanoparticle sizes did not change for a duration of 30 days on the shelf in the dark.

We verified the electron transfer from P3H‐T to F_4_TCNQ, and hence the electrostatic stabilization by electrical doping, by UV–VisNIR absorption spectrometry. Figure 2b depicts the normalized absorbance of the dispersions on a logarithmic scale to better visualize the P3HT polaron (700 – 1000 nm),^[^ ^17^, ^18^ ^]^ the F_4_TCNQ^−^ anion (410, 750, and 855 nm),^[^ ^19^ ^]^ and the F_4_TCNQ^2−^ dianion (332 nm).^[^ ^19^ ^]^ For reference, the absorbance of a P3HT solution in chloroform is depicted. The bathochromic shift from solution to dispersion (i.e., the red shift of the absorption maximum) and the emergence of additional absorption bands indicate the solidification of P3HT.^[^ ^20^, ^21^ ^]^

While previous studies on the electrostatic stabilization of P3HT dispersions in alcohol demonstrated that the charging of P3HT occurs by transfer of one electron to each F_4_TCNQ, forming only F_4_TCNQ^−^ anions, we observed different transfer dynamics in aqueous dispersions. In all dispersions, we observed the P3HT polaron absorbance increasing with the concentration of F_4_TCNQ, indicating a gradual increase of the charge density on P3HT. However, we did not observe signatures of the F_4_TCNQ^−^ anion at concentrations *ζ*_F4TCNQ_ < 5 wt%, but found the emergence of the signature of F_4_TCNQ^2−^ dianions instead. Only at *ζ*_F4TCNQ_ ≥ 5 wt%, we did observe strong evidence for the formation of F_4_TCNQ^−^ anions. In accordance with the literature,^[^ ^22^, ^23^, ^24^ ^]^ we conclude that matrix effects, stemming from the dispersion medium water, promote the formation of F_4_TCNQ^2−^ at small *ζ*_F4TCNQ_ (Figure S3, Supporting Information). The ionization potentials of semiconductors depend on their ionization potential in vacuum and the polarization energy of the environment. Thus, the high permittivity of water (*ε*_r_ = 80.1)^[^ ^25^ ^]^ leads to an energy level shift of the P3HT upon nanoprecipitation, which promotes the formation of the F_4_TCNQ^2−^ dianion. Toward higher concentrations, the thermodynamic equilibrium of the doping process is shifted toward the formation of F_4_TCNQ^−^. We note that at all concentrations *ζ*_F4TCNQ_, F_4_TCNQ^−^ is the prevailing counterion in THF solution before nanoprecipitation (Figure S3a, Supporting Information), whereas no F_4_TCNQ^2−^ occurs, because the less polar THF induces less polarization into the doping process. We also note that the absorbance of the dispersion with *ζ*_F4TCNQ_ = 0.5 wt% seemingly does not follow this trend but exhibits a much broader spectral distribution. With respect to the much larger nanoparticle sizes of 180 nm, this effect is likely to originate from light scattering and hence enhanced extinction.

In order to enable net charging of the nanoparticles and hence to contribute to the stability of the dispersion, the F_4_TCNQ^−^ anions and the F_4_TCNQ^2−^ dianions must detach from the nanoparticles.^[^ ^9^ ^]^ According to the absorbance measurements in Figure S3a (Supporting Information), the electron transfer from P3HT to F_4_TCNQ occurs already in THF solution, and F_4_TCNQ^−^ is formed before nanoprecipitation in water. In THF solution, the F_4_TCNQ^−^ adheres to the P3HT^+^ by coulombic forces, as these are not shielded by the low permittivity of THF (*ɛ*_r_ = 7.52^[^ ^25^ ^]^). Water has a permittivity of *ε*_r_ = 80.1, which promotes separation of the negatively charged F_4_TCNQ^−^ ions in the dispersion even more strongly than, for example, the dispersion medium ethanol (*ε*_r_ = 25.3)^[^ ^9^, ^25^ ^]^ and thus may explain the excellent long‐term stability of the aqueous dispersions. This conclusion is supported by an enhancement of the residual solubility of F_4_TCNQ upon charging, which facilitates the detachment of the F_4_TCNQ^−^ counterions from the nanoparticles after charge transfer. Since the solubility of F_4_TCNQ in water is not accessible experimentally as it would gradually turn into F_4_TCNQ^−^/F_4_TCNQ^2−^, we assessed it with density functional theory simulations using the Amsterdam Density Functional software suite, Solvation Model 12. Table S1 (Supporting Information) summarizes the simulation results. The negative solvation energy of the F_4_TCNQ^−^ anion in water has a higher magnitude than the solvation energy of the neutral molecule, thus improving the solubility after charge transfer to the dopant and thus facilitating the separation of F_4_TCNQ^−^ anions from the P3HT^+^.

1,3,4,5,7,8‐hexafluorotetracyanonaphthoquinodimethane (F_6_TCNNQ) is a dopant with a nominally larger electron affinity than F_4_TCNQ. Yet, in this study, we found that F_6_TCNNQ is less efficient in attracting electrons from P3HT than F_4_TCNQ (Figure S4, Supporting Information) which may again be related to interaction with the matrix which is why we have discarded F_6_TCNNQ for further studies.

### 2.2 Stabilization of P3HT:Fullerene Nanoparticles in Water

After optimizing the F_4_TCNQ concentrations to ζ_F4TCNQ_ = 2.5 wt% for the best stabilization of the P3HT nanoparticle dispersions and smallest nanoparticle sizes, we completed the BHJ by adding either of the fullerenes IC_60_BA or PC_71_BM. Both fullerenes nest well into P3HT and thus can be passively stabilized by the electrostatic charging of P3HT.^[^ ^12^ ^]^ Both, P3HT and either of the fullerenes were dissolved separately in THF, mixed (1:1 w/w), and then subdued to the nanoparticle dispersion synthesis described above, yielding a total semiconductor concentration in the dispersion of c_S_ = 1 g L^−1^. The P3HT:PC_71_BM nanoparticles initially exhibited a mean size of 104 nm (Figure S5a, Supporting Information). Notably, the aqueous nanoparticle dispersions showed remarkable long‐term stability despite the omission of the commonly used surfactants: after one day on the shelf, the nanoparticle size did not change within the measurement precision. And even after more than 70 days the dispersion stability prevailed. Seemingly smaller P3HT:PC_71_BM nanoparticle sizes of 94 nm may stem from either precipitated larger nanoparticles or agglomerates of nanoparticles, both of which change the size distribution in the measurement and hence the mean nanoparticle size. Likewise, the initial P3HT:IC_60_BA nanoparticle size was 90 nm which decreased to 72 nm after 70 days. The UV–Vis‐NIR absorbance spectra of the dispersions (Figure S5b, Supporting Information) exhibit signatures of both the P3HT (400–600 nm) and the fullerenes (<400 nm), indicating the incorporation of both semiconductors. In both dispersions, the polaron band is visible in the spectral regime from 700 to 1000 nm. The enhanced electrostatic stabilization of dispersions synthesized in the presence of F_4_TCNQ is also evident in the zeta potential measurements which we performed on P3HT:PC_71_BM and P3HT:IC_60_BA dispersions with ζ_F4TCNQ_ = 2.5 wt%. We found an electrophoretic mobility of µ_E_ = −3.4∙10^‒8^ m^2^ V^−1^ s^−1^ of the P3HT:PC_71_BM nanoparticles, resulting in a zeta potential of z = ‒43 mV. The P3HT:IC_60_BA nanoparticles exhibited an electrophoretic mobility of µ_E_ = −3.1∙10^−8^ m^2^ V^−1^ s^−1^ and thus a zeta potential z = −44 mV. Dispersions are considered stable if their zeta potential exceeds z = ±30 mV, but the sign of the zeta potential does not allow conclusions on the sign of the nanoparticle charge.^[^ ^26^ ^]^

### 2.3 Hydrogen Generation

To demonstrate the photocatalytic activity of the nanoparticles, we opted for aqueous P3HT:PC_71_BM (1:1 w/w) dispersions stabilized with F_4_TCNQ (*ζ*_F4TCNQ_ = 2.5 wt%) which showed a better nanoparticle size consistency over time than P3HT:IC_60_BA dispersions as described above. In order to ensure rather homogenous irradiation throughout the dispersion volume for a better quantitative analysis, the semiconductor concentration was reduced to *c*_S =_ 0.05 g L^−1^.

**Figure 3:**
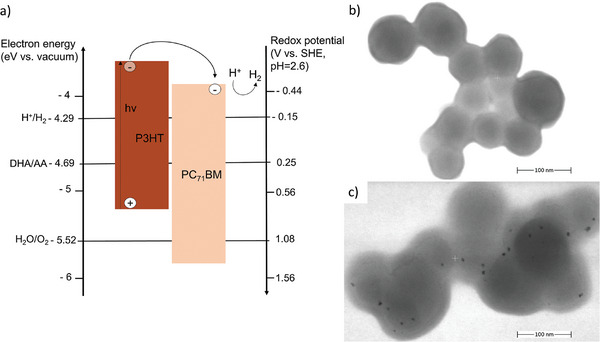
a) The energy scheme of the photocatalytic system shows the redox potentials versus a standard hydrogen electrode (SHE) of the hydrogen evolution reaction (H^+^/H_2_), the oxygen evolution reaction (H_2_O/O_2_) and the sacrificial reagent ascorbic acid (DHA/AA, c_AA_ = 0.1 mol L^−1^, two‐hole oxidation) at pH = 2.6^[^ ^27^ ^]^ as well as the IPs and EAs of P3HT and PC_71_BM, the latter two of which together form the BHJ within the nanoparticles. b) STEM image of a cluster of P3HT:PC_71_BM nanoparticles without the platinum co‐catalyst and c) with the platinum co‐catalyst attached (dark spots).

**Figure** **3**a depicts the energy levels of all compounds involved in hydrogen generation. Electron‐hole pairs are photogenerated on P3HT and then dissociated by electron transfer to PC_71_BM. The electron affinity (EA) of PC_71_BM is energetically well‐situated to enable proton reduction. Yet, the ionization potential (IP) of P3HT (*E*_IP_ = −5.2 eV) is too shallow to promote the oxygen evolution reaction. Therefore, ascorbic acid (AA, *c*_AA_ = 0.1 mol L^−1^) was added to the dispersion as a sacrificial reagent to regenerate the photogenerated holes in the P3HT through the formation of dehydroascorbic acid (DHA, with the potential DHA/AA at −4.69 eV versus vacuum at pH = 2.6).^[^ ^27^ ^]^ The co‐catalyst platinum (*ζ*_Pt_ = 5 wt%) was attached to the nanoparticles by converting the precursor hexachloroplatinic acid (H_2_PtCl_6_) through photodeposition.^[^ ^28^ ^]^ In Figure 3b, a bright‐field (BF) scanning transmission electron microscopy (STEM) image of a representative nanoparticle assembly is depicted, drop cast from the platinum‐free dispersion on a transmission electron microscopy grid. After the photodeposition process, in Figure 3c, the nanoparticles feature small dark spots. As the electrons of the scanning beam are scattered by heavy metals away from the BF‐STEM detector segment, we interpret these small dark features on the nanoparticle surface as platinum deposits. We note that the seemingly larger nanoparticles in Figure 3c are an effect of the electron beam on the polymer nanoparticles during STEM measurements (beam induced contamination), and their size may also be affected by residues of ascorbic acid. The micrograph is focused on the platinum nanoparticles which involves more frequent scanning, upon which swelling of the organic nanoparticles occurs, resulting in seemingly larger nanoparticles.

After completion of the catalytic setup, that is P3HT:PC_71_BM nanoparticles with platinum co‐catalyst attached and stabilized with F_4_TCNQ, we recorded photoluminescense spectra of the nanoparticle dispersions, showing quenching of the photoexcited electron‐hole pairs by both the fullerene and the platinum (Figure S6, Supporting Information).

To measure the hydrogen generation, we utilized a MQ8 hydrogen sensor coupled to an Arduino microcontroller. MQ8 hydrogen sensors are designed to measure hydrogen concentrations beyond 100 ppm. The sensor was then inserted into the vial that contains the dispersion with the aid of a snap‐on lid. The MQ8 resistively measured the hydrogen concentration in the gas volume of the vial. No substances to which the sensor has cross‐sensitivity (alcohol, CO, CH_4_, liquid petroleum gas), were in the vicinity. The Arduino recorded the measurement data and calculated the hydrogen content in the enclosed gas volume using the script which is provided in the Supporting Information. A chip‐on‐board LED (COB‐LED) is used to illuminate the setup and to start the photocatalytic reaction. For a photo of the setup and the illumination spectrum, see Figure S7 (Supporting Information). Notably, the costs of the components sum up to only 25 €.

**Figure 4:**
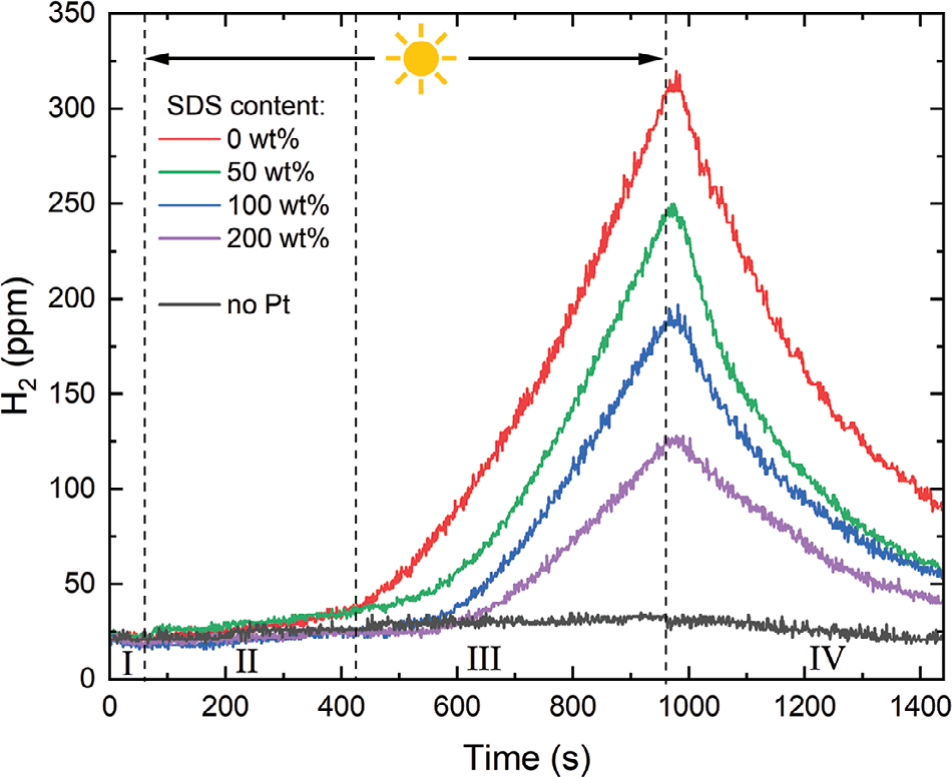
Hydrogen evolution in electrostatically stabilized P3HT:PC_71_BM nanoparticle dispersions (red line) in the dark (regime I), under irradiation (regime II+III), and after switching off the light (regime IV). Irradiation of the setup first triggers the photodeposition of the platinum co‐catalyst onto the nanoparticle surface in regime II and then drives the hydrogen generation in regime III. The addition of the stabilizing surfactant SDS (50, 100, and 200 wt% vs. semiconductor mass) deteriorates the hydrogen evolution rate (green, blue, and purple lines). For reference, we also investigated a dispersion without co‐catalyst (black line) that did not produce any hydrogen.

**Figure** **4** shows the hydrogen evolution over time in a P3HT:PC_71_BM dispersion under irradiation. The graph is divided into four operational regimes. In the beginning, in regime I, the dispersion is placed in the dark, and the corresponding measurement signal of the MQ8 hydrogen sensor shows a constant hydrogen concentration of 23 ppm. Since the hydrogen content in the air is 0.6 ppm,^[^ ^29^ ^]^ this baseline measurement represents the lower detection limit of the setup. After 60 s, the dispersions were irradiated with the COB‐LED. In regime II, we detected no hydrogen evolution. During this time, we suspect the platinum deposition to occur, triggered by irradiation, completing the photocatalysis setup. 420 s after the start of the experiment, in regime III, we observed a steep increase of the hydrogen content in the gas volume under continuous irradiation (red line), which demonstrates the photocatalytic activity of the electrostatically stabilized dispersions. After 960 s (i.e., after 15 min of irradiation), the COB‐LED was switched off again which is denoted as regime IV. The diminishing hydrogen concentration in regime IV confirms the photocatalytic hydrogen generation that quickly abates in the dark. The exponential decay can be attributed to the loss of hydrogen from the enclosed volume which was not sufficiently tight for hydrogen. Likewise, oxygen in the vial may have promoted the back‐reaction from hydrogen to water. When repeating the experiment after 13 h of illumination, we found the same qualitative hydrogen evolution in the dispersion, however, at a somewhat smaller magnitude (Figure S8, Supporting Information). For reference, we also tested the same dispersion without the addition of the platinum co‐catalyst, but did not see any hydrogen evolution beyond the baseline (black line). Minor fluctuations of the hydrogen generation rate may stem from changes in temperature or humidity within the gas sample volume.

This observation of continuous hydrogen evolution lets us to conclude that the electrostatic stabilization of the nanoparticle dispersion, remarkably, is strong enough to persist the charge transfer between the nanoparticles, the co‐catalyst, and the aqueous dispersion medium during hydrogen evolution, opening up a new process design for future photocatalytic reactions in absence of stabilizing surfactants.

In order to compare the hydrogen evolution in the electrostatically stabilized dispersions with organic nanoparticle dispersions which are stabilized with surfactants, we added sodium dodecyl sulfate (SDS) to our dispersions in different amounts after synthesis. For the best comparison, we have deliberately chosen to add SDS to the dispersions that were synthesized by nanoprecipitation and stabilized electrostatically. This approach allows the direct comparison of nanoparticles with identical micromorphology so that SDS can only impact the photocatalytic nanoparticle surface. For best comparison, we used about the same amount of surfactant that is commonly used for state‐of‐the‐art synthesis of surfactant‐stabilized dispersions.^[^ ^30^, ^31^ ^]^ Often, surfactants are partly removed from the dispersion after synthesis,^[^ ^30^ ^]^ but the literature is vague about the remaining concentration. We have investigated a series of dispersions with surfactant concentrations of 50 wt% (vs. semiconductor mass, green line), 100 wt% (blue line), and 200 wt% (purple line) to demonstrate the principal effect of surfactants on hydrogen generation. As depicted in Figure 4, the hydrogen generation rate is significantly reduced toward increasing amounts of SDS in otherwise identical nanoparticle dispersions. This clearly shows that surfactants are indeed detrimental to the photocatalytic process and should be omitted whenever possible.

## 3 Conclusion

We have synthesized long‐term stable and surfactant‐free aqueous nanoparticle dispersions of P3HT, P3HT:PC_71_BM, and P3HT:IC_60_BA using nanoprecipitation. The nanoparticles were charged by electron transfer to the strong molecular acceptor F_4_TCNQ, promoting electrostatic stabilization, and successfully translating a stabilization mechanism that was only deployed in dispersions in alcohols or acetonitrile before.

The aqueous P3HT:PC_71_BM dispersions were then used for photocatalytic hydrogen generation. For this purpose, platinum was photodeposited on the nanoparticle surface from H_2_PtCl_6_. The electrostatic stabilization of the nanoparticle dispersion did not perish upon the photodeposition of platinum or photocatalysis, which is remarkable as photocatalysis involves many charge‐transfer reactions. The addition of SDS to the dispersion, which is one of the surfactants commonly used to stabilize state‐of‐the‐art organic nanoparticle dispersions for hydrogen generation, led to a significant reduction of the photocatalytic activity. This finding highlights the importance of the surfactant‐free stabilization of dispersions for maximum hydrogen evolution rates and points a promising pathway toward highly efficient photocatalysis in nanoparticle dispersions in the future.

## 4 Experimental Section

All experiments were performed in a class 10,000 cleanroom.

### Materials

Regioregular P3HT (“4002‐EE”, M_w_^ =^ 50‐70 kg mol^−1^, regioregularity > 90%) was purchased from Rieke Metals, IC_60_BA and PC_71_BM from Lumtec. All organic semiconductors were used without additional purification. F_4_TCNQ was purchased from Ossila and F_6_TCNNQ from 1‐Material. Hexachloroplatinic acid and ascorbic acid were supplied by Merck. SDS was supplied by Acros Organics. THF and acetonitrile (analytical grade) were purchased from Merck. Milli‐Q water was used for all experiments.

### Nanoparticle Synthesis

P3HT and the fullerenes were dissolved separately in THF (total semiconductor concentration *c*_S_ = 2 g L^‒1^) under stirring on a hotplate (47 °C) for at least 30 min. The blend solution was prepared by mixing the P3HT and fullerene solutions (1:1 w/w). THF was added to the blend solution to adjust the semiconductor concentration in the solution to *c*_S_ = 1 g L^−1^. The strong molecular acceptor F_4_TCNQ (dopant) was first dissolved separately in acetonitrile (*c*_F4TCNQ_ = 10 g L^−1^) and then added to the blend solution in the desired quantity. To avoid pipetting inaccuracies when needing smaller F_4_TCNQ quantities (*ζ*_F4TCNQ_ *= *0.5 wt% or 1 wt%), the F_4_TCNQ solution was diluted (*c*_F4TCNQ_ = 1 g L^−1^) with acetonitrile. For the nanoprecipitation process, the (doped) P3HT:fullerene solution (THF, 1 mL) was injected into a beaker with the non‐solvent water (3 mL) under vigorous stirring using a pipette at room temperature (20 °C) and under irradiation by a COB‐LED (30 W, 1 A).^[^ ^10^ ^]^ After nanoprecipitation, the solvent THF was evaporated, and the water volume was reduced in a water bath (70 °C) to restore the original semiconductor concentration (*c*_S_ = 1 g L^−1^).

### Hydrogen Evolution

A vial was primed with 1,700 µL of Milli‐Q water. Then the P3HT:PC_71_BM dispersion (100 µL, *c*_S_ = 1 g L^−1^) and aqueous ascorbic acid solution (200 µL, 1 m) as sacrificial reagent were added (total volume 2 mL), producing a semiconductor concentration of 0.05 g L^−1^ and an ascorbic acid concentration of 0.1 m. An aqueous hexachloroplatinic acid solution (5 µmol L^−1^) was added to the dispersion as a precursor for the platinum co‐catalyst. Where indicated, an aqueous solution of SDS (*c*_SDS_ = 10 g L^−1^) was added in suitable amounts. The dispersion was illuminated from the side with the COB‐LED (93 W, 1,200 W m^−2^, spectrum Figure S7, Supporting Information), first to trigger the conversion of hexachloroplatinic acid to platinum and then to start the hydrogen generation. The photogenerated hydrogen was measured simultaneously with a MQ8 hydrogen sensor and recorded with an Arduino (see Supporting Information for details).

### Nanoparticle Size Measurements

The intensity‐based mean nanoparticle size (hydrodynamic diameter) of the dispersions was determined by dynamic light scattering (DLS, Zetasizer Nano ZS, Malvern Panalytical, 20 °C, dynamic viscosity 1.0031 mPa s^−1^, refractive index 1.330) in quartz cuvettes following standard measurement protocols. The stated nanoparticle sizes correspond to the average of 12 repeating measurements of each sample. The error bars represent the data range (min/max). For each sample, an aliquot of the stock dispersion was diluted with water (20 µL dispersion with *c*_S =_ 1 g L^−1^, diluted in 1.5 mL water).

### Zeta‐Potential Measurements

The electrophoretic mobility and the zeta potential were measured by electrophoretic light scattering (Zetasizer Nano ZS, Malvern Panalytical). Therefore, the same semiconductor concentrations were used as for the DLS measurements (0.02 g L^−1^) by diluting the dispersions in Milli‐Q water. The measurements were carried out in electrophoretic “dip” cells (Malvern Panalytical).

### Absorbance Measurements

The absorbance of the dispersions was measured in two‐ray transmission mode employing quartz cuvettes (width 1 cm) in a spectrophotometer (Cary5000, Agilent Technologies), using the same diluted dispersions that had been utilized for the nanoparticle size determination by DLS. The absorbance was baseline‐corrected by accounting for the absorbance of a reference cuvette containing pure water.

### Scanning Transmission Electron Microscopy

The nanoparticle dispersions were drop cast onto a copper TEM grid. A FEI Helios G4 FX was used to record STEM images at 350,000× magnification with the bright‐field detector at an acceleration voltage of *V*_acc_ = 30 kV.

### Statistical Analysis

In Figure 2a, each data point represents the mean nanoparticle size derived from 12 measurements of the same sample. The error bars show the corresponding data range (min/max). The as‐recorded absorption spectra in Figure 2b are baseline‐corrected and normalized to the P3HT absorption peak. The STEM images in Figure 3b,c are depicted as recorded without further image processing. The hydrogen concentration data in Figure 4 is plotted as received from the Arduino microcontroller, measured on representative individual samples.

## Conflict of Interest

The authors declare no conflict of interest.

## Supporting information

Supporting Information

## Data Availability

The data that support the findings of this study are available from the corresponding author upon reasonable request.
