# Supplementary material for: Photocatalytic Hydrogen Generation in Surfactant‐Free, Aqueous Organic Nanoparticle Dispersions
Source: Small. 2024 Oct 2;20(47):2406236. doi: 10.1002/smll.202406236 (PMC11579955; doi:10.1002/smll.202406236)
Supplement: Supplementary file 1 — Supporting Information [file SMLL-20-2406236-s001.pdf]

NANO · MICRO
small

Supporting Information

for *Small*, DOI 10.1002/smll.202406236

Photocatalytic Hydrogen Generation in Surfactant-Free, Aqueous Organic Nanoparticle Dispersions

*Jan Bruder, Karen Fischer, Jonas Armleder, Erich Müller, Nicola Da Roit, Silke Behrens, Yuman Peng, Wolfgang Wenzel, Holger Röhm and Alexander Colsmann**

Supporting Information

Photocatalytic Hydrogen Generation in Surfactant-Free, Aqueous Organic Nanoparticle Dispersions

*Jan Bruder, Karen Fischer, Jonas Armleder, Erich Müller, Nicola Da Roit, Silke Behrens, Yuman Peng, Wolfgang Wenzel, Holger Röhm, Alexander Colsmann**

Karlsruhe Institute of Technology (KIT), Germany

Keywords: photocatalysis, hydrogen generation, organic nanoparticle dispersions, aqueous dispersions, surfactant-free

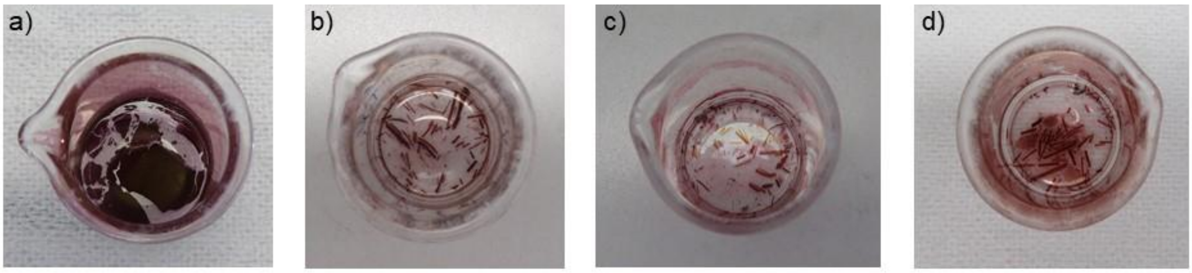

Figure S1. Sediments after synthesis of P3HT dispersions in water by nanoprecipitation from THF solution and subsequent volume reduction. (a) If no F_4TCNQ is used to stabilize the dispersion, patches of P3HT form which remain in the beaker. Only smallest amounts of P3HT remain in dispersion (Figure S2a). (b) Upon addition of F_4TCNQ, stable dispersions form. Still, some material may sediment in the beaker, yet without changing the fundamental properties of the dispersion. This effect occurs independent of the F_4TCNQ concentration ζ_{F4TCNQ} and can be attributed to F_4TCNQ-induced crystallization of P3HT.[S1] (c) The same effect occurs if F_6TCNNQ is used to charge the nanoparticles or (d) if fullerenes are blended into P3HT.

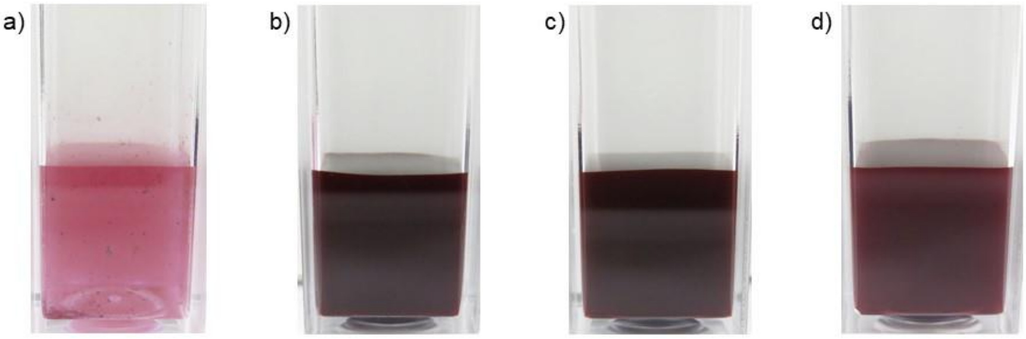

Figure S2. Photos of aqueous P3HT dispersions after synthesis by nanoprecipitation from THF solution, subsequent volume reduction and removal of the P3HT slabs (Figure S1). (a) If F_4TCNQ is omitted, the P3HT load in the dispersion is low and large agglomerates of P3HT are visible. (b) Upon the addition of F_4TCNQ (2.5 wt%), the optical density of the P3HT dispersion is enhanced, indicating a much higher semiconductor load in the dispersion. The dispersion remains stable over several months. (c,d) The same effect was observed on dispersions comprising P3HT:PC_{71}BM with F_4TCNQ (2.5wt%) or P3HT:IC_{60}BA with F_4TCNQ (2.5wt%).

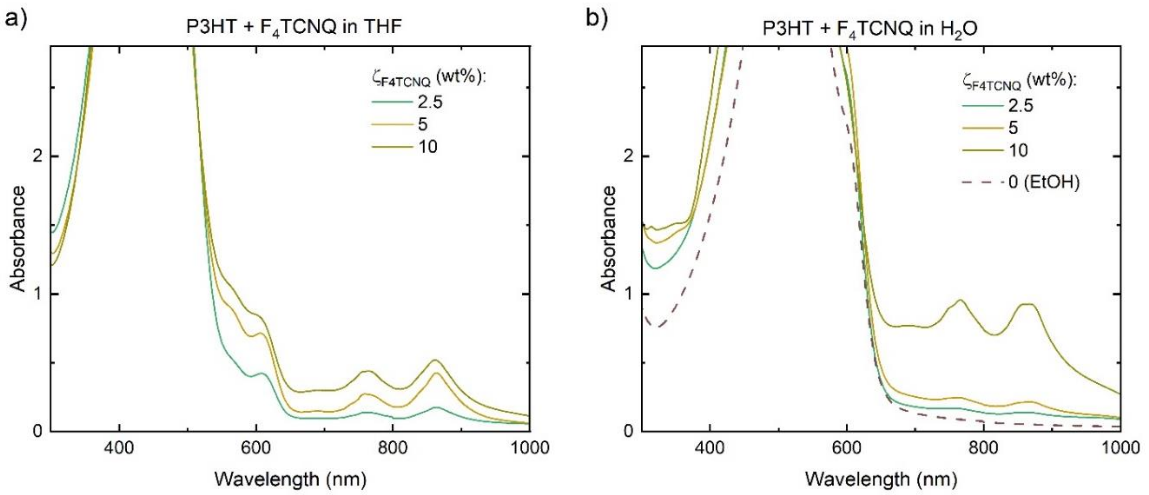

Figure S3. UV-Vis-NIR absorbance of P3HT doped with F_4TCNQ, recorded in solution before nanoprecipitation and in dispersion after nanoprecipitation. High concentrations of the solutions and dispersions were chosen in order to better resolve the characteristic absorption of the F_4TCNQ^- anion, however, leading to a saturation of the P3HT absorption peak. (a) Absorbance in THF solution before nanoprecipitation: the characteristic absorption peaks of the F_4TCNQ^- anion appear at 760 nm and 860 nm. (b) Absorbance in H_2O dispersion after evaporation of the THF: at low concentrations, the signatures of the anion are deteriorated (ζ_{F4TCNQ} = 2.5 wt% and 5 wt%). Instead, the characteristic absorption of the F_4TCNQ^{2-} dianion becomes visible at 332 nm. For reference, the graph also shows a P3HT dispersion with ζ_{F4TCNQ} = 0 wt%, however, this dispersion was synthesized in ethanol (EtOH) since P3HT dispersions in water are not stable without the addition of F_4TCNQ.

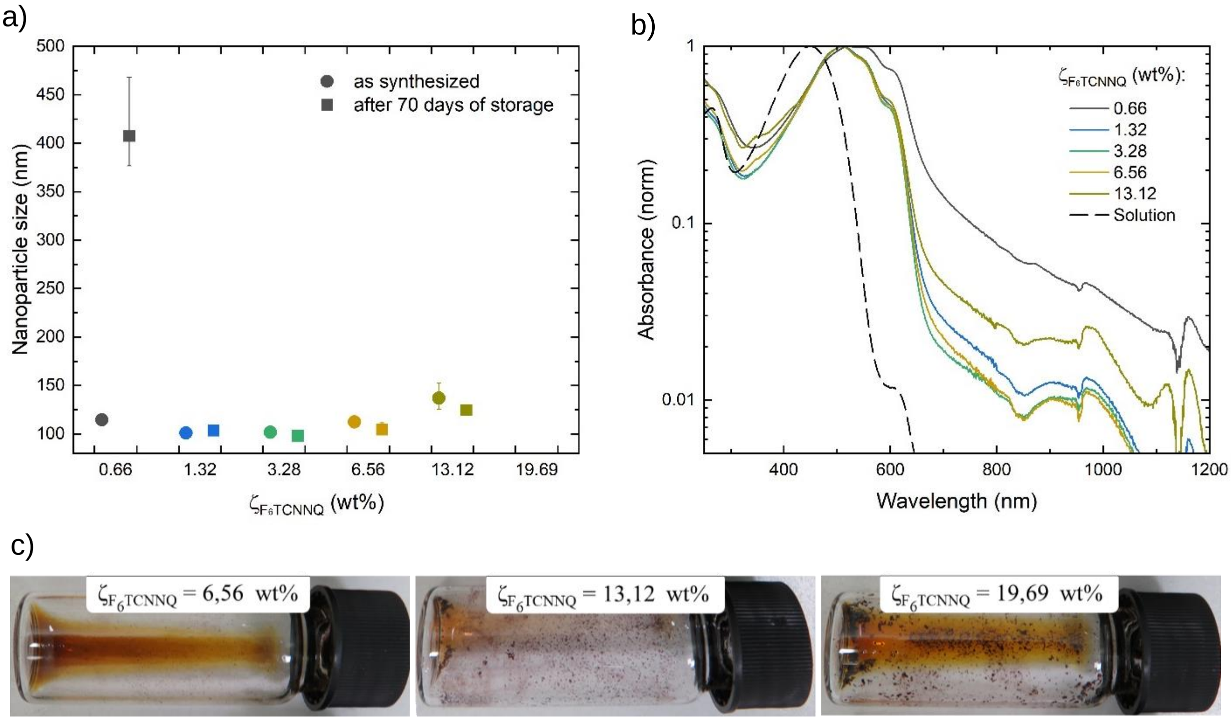

Figure S4. Properties of P3HT nanoparticle dispersions charged and stabilized with F_6TCNNQ instead of F_4TCNQ. (a) Nanoparticle sizes in dependence of the F_6TCNNQ concentration $\zeta_{F6TCNNQ}$. $\zeta_{F6TCNNQ}$ was adjusted to match the molar concentrations of F_4TCNQ in the other experiments. For $\zeta_{F6TCNNQ}$ = 1.32 wt% and $\zeta_{F6TCNNQ}$ = 3.2 wt%, we found the smallest nanoparticles with a diameter of 100 nm. Less or more of F_6TCNNQ produced larger nanoparticles which is very much in line with the observations on the F_4TCNQ-stabilized dispersions. The nanoparticles also achieved excellent long-term stability, as demonstrated by the nanoparticle size measurements after 70 days of storage (10 weeks). (b) In contrast to the stabilization with F_4TCNQ, the P3HT polaron band (700 – 1000 nm) is less notable in the absorbance of the P3HT dispersions at different $\zeta_{F6TCNNQ}$. (c) Already before nanoprecipitation, we found some visible P3HT aggregates in F_6TCNNQ-doped solutions, but this did not affect the process. However, for $\zeta_{F6TCNNQ}$ = 19.69 wt% and beyond, the agglomeration became so strong that stable dispersions could no longer be achieved.

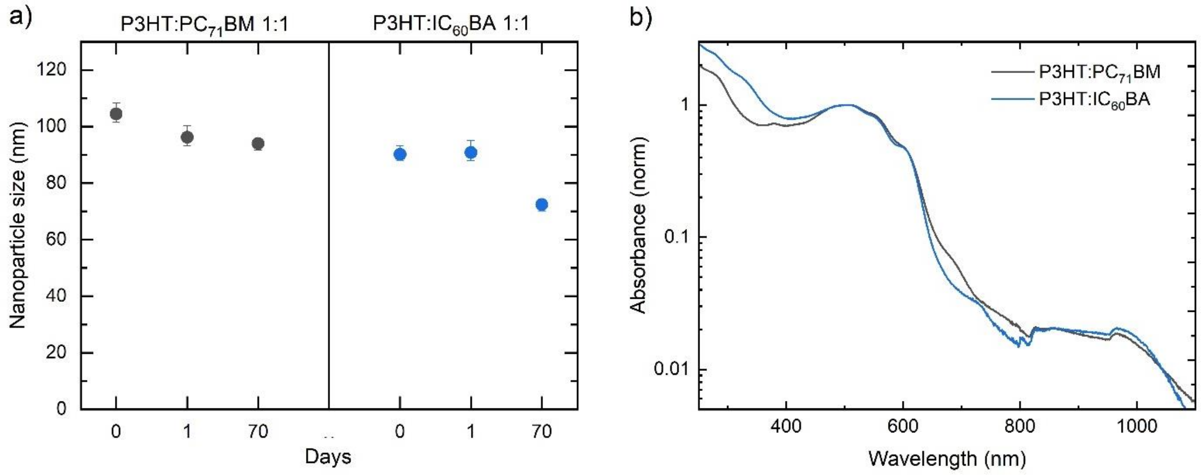

Figure S5. (a) Nanoparticle sizes of P3HT:$IC_{60}BA$ and P3HT:$PC_{71}BM$ dispersions (both 1:1 w/w) stabilized with F_4TCNQ (ζ_{F4TCNQ} = 2.5 wt%). The nanoparticle sizes were measured by dynamic light scattering (DLS) right after synthesis, after 1 day and after 70 days (10 weeks) on the shelf. The error bar shows the data range (min/max) of 12 repeating measurements of the same dispersions. (b) UV-Vis-NIR absorbance spectra of the dispersions normalized to the P3HT peak.

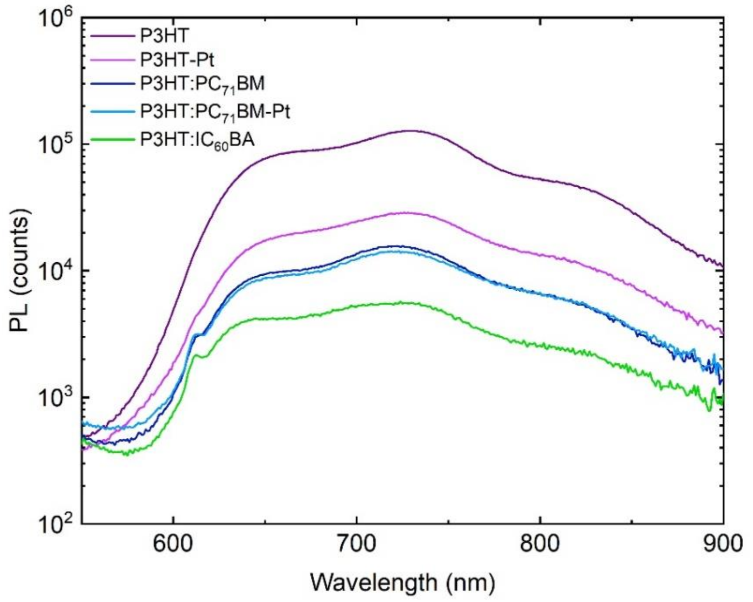

Figure S6. Photoluminescence (PL) spectra of P3HT and P3HT:$PC_{71}BM$ dispersions, all doped with F_4TCNQ (2.5 wt%). The PL quenching by $PC_{71}BM$ indicates a well intermixed bulk-heterojunction inside the nanoparticles. Likewise, the photodeposited platinum quenches the PL of P3HT. The quenching effect of platinum on the P3HT:$PC_{71}BM$ dispersions is negligible. Strong coagulation during platinum photodeposition occurs on P3HT:$IC_{60}BA$ dispersions making meaningful PL measurements impossible.

The photoluminescence was measured with a spectrofluorometer (FS5, Edinburgh Instruments) with the cuvette placed in the front face sample holder (module SC-10). The stock dispersion (after volume reduction) was diluted with water (100 µL of dispersion in 3.9 mL of water, concentration of semiconductor after dilution 25 mg L^{-1}). The platinum-containing dispersion was diluted with water to yield the same semiconductor concentration. The samples were excited at 500 nm (slit width 6 nm) and the emission spectrum was measured from 550 to 900 nm (slit width 2 nm).

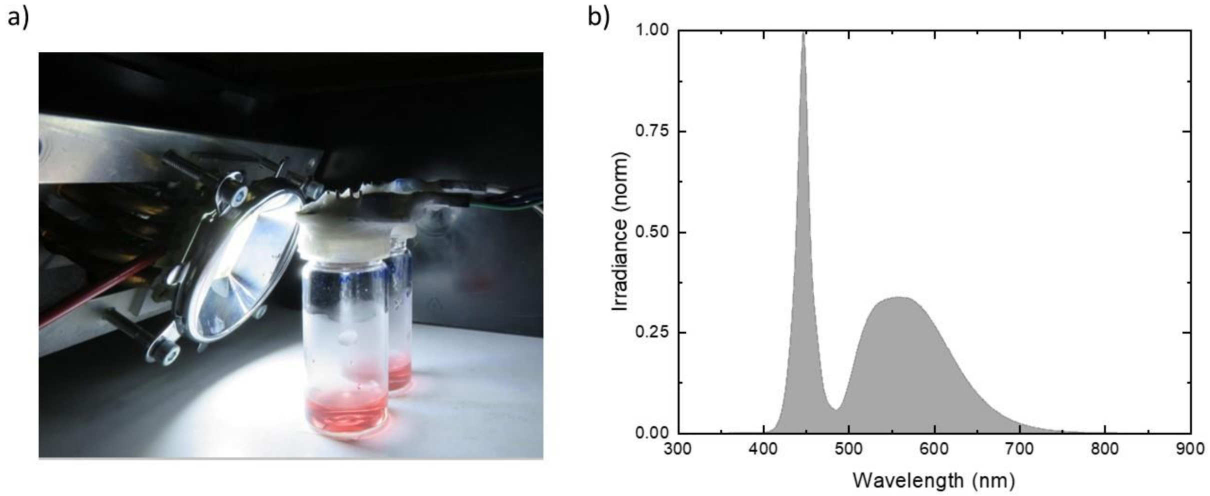

Figure S7. Measurement setup for hydrogen detection. (a) A COB-LED is used to illuminate the the dispersions. The MQ8 hydrogen sensors, which were connected to an Arduino microcontroller board, were mounted on top. The Arduino source code is provided at the end of this document. (b) Spectrum of the COB-LED (1200 W m^{-2}) on the samples.

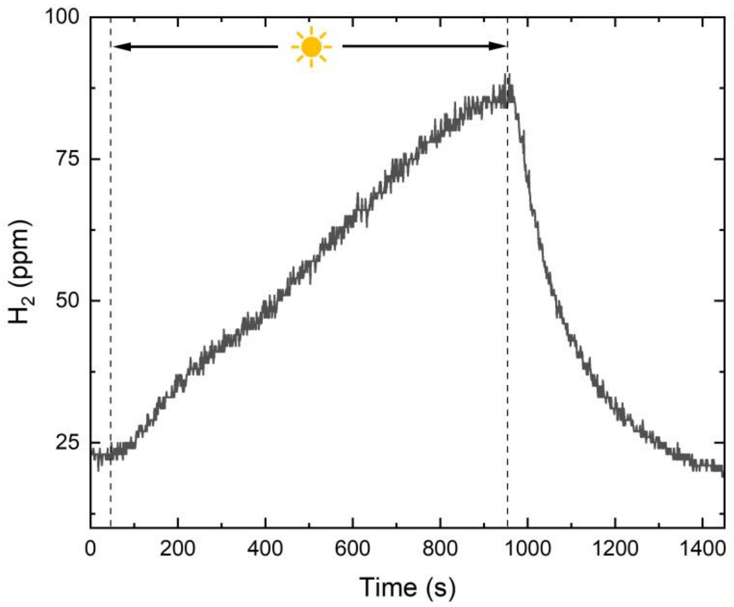

Figure S8. When repeating the hydrogen generation experiment after 13 h of illumination, we found the same qualitative hydrogen evolution in the P3HT:$PC_{71}BM$ dispersion (2.5 wt% F_4TCNQ). Yet, the photocatalytic activity has decreased compared to the initial measurement (Figure 4 of the main manuscript).

	Solvation energy in water, eV			Solvation energy in acetonitrile, eV		
	G_p	G_{cds}	G	G_p	G_{cds}	G
Neutral F_4TCNQ	-0.77	0.31	-0.46	-0.75	0.28	-0.47
F_4TCNQ^- anion	-1.96	0.31	-1.65	-1.92	0.28	-1.64

Table S1. Computed solvation energies of neutral F_4TCNQ and F_4TCNQ^- anions in water. All calculations were conducted using the Amsterdam Density Functional (ADF) software suite. Initially, we optimized the geometry of the solute molecule F_4TCNQ in the gas phase employing density functional theory (DFT). The exchange-correlation interactions were modeled using the hybrid B3LYP functional [S2] with the TZ2P [S3] basis set. Subsequently, the solvation free energies of F_4TCNQ from the gas phase to the solution phase were estimated using the generalized Born solvation model SM12.[S4] This calculation utilized the geometry optimized in the gas phase. Solvation parameters, such as the dielectric constant of the solvent, were determined by specifying the solvent name in the SOLVATION SM12 key block.[S5] Further details on these relevant parameters can be found in previous reports.[S4]

In the SM12 model, the total solvation energy G is the sum of the polarization free energy of the solute–solvent system G_p when the solute is inserted, and the free energy that is nominally associated with cavitation, dispersion, and solvent structure G_{cds}.

For reference, we also calculated the solvation energy of F_4TCNQ in acetonitrile from which the F_4TCNQ is introduced into the dispersion (solubility of acetonitrile: $c_{ACN} > 10$ g L^{-1}). Physically, the similar solvation energies of the anion can be understood in context of the high polarity of both solvents. The F_4TCNQ anion features four –C≡N groups, which may contribute to the dispersion of the charge and reduce the dependence on solvent specificity, resulting in a similar energy in both polar solvents, water and acetonitrile.

Source code for Arduino hydrogen generation

The original source code was obtained from [S6]. For this work, it was expanded to include a second sensor to enable the simultaneous measurement of a reference.

```
/**********************Application Related Macros**********************************/

#define       GAS_H2          (0)

/*****************************Globals***********************************************/

float         H2Curve[3]  =  {2.3, 0.93,-1.44};   //two points are taken from the curve in datasheet.
                                                  //with these two points, a line is formed which is
                                                  //"approximately equivalent to the original curve.
                                                  //data format:{ x, y, slope}; point1: (lg200, lg8.5),
                                                  //point2: (lg10000, lg0.03)
float         Ro1         =  10;                  //Ro1 is initialized to 10 kilo ohms
float         Ro2         =  10;                  //Ro2 is initialized to 10 kilo ohms

void setup(){
  Serial.begin(9600);                             //UART setup, baudrate = 9600bps
  Serial.print("Calibrating...\n");
  Ro1 = MQCalibration(MQ_PIN1);                   //Calibrating the sensor. Please make sure the sensor
                                                  //is in clean air when you perform the calibration
  Ro2 = MQCalibration(MQ_PIN2);
  Serial.print("Calibration is done...\n");
  Serial.print("Ro1=");
  Serial.print(Ro1);
  Serial.print("Ro2=");
  Serial.print(Ro2);
  Serial.print("kohm");
  Serial.print("\n");
}

void loop(){
   Serial.print("H2:");
   Serial.print(MQGetGasPercentage(MQRead(MQ_PIN1)/Ro1,GAS_H2));
   Serial.print( "ppm  ");
   Serial.print(MQGetGasPercentage(MQRead(MQ_PIN2)/Ro2,GAS_H2));
   Serial.print( "ppm");
   Serial.print("\n");
   delay(200);
}
/****************** MQResistanceCalculation ****************************************
Input:   raw_adc - raw value read from adc, which represents the voltage
Output:  the calculated sensor resistance
Remarks: The sensor and the load resistor forms a voltage divider. Given the voltage across the load
resistor and its resistance, the resistance of the sensor could be derived.
************************************************************************************/
float MQResistanceCalculation(int raw_adc){
  return (((float)RL_VALUE*(1023-raw_adc)/raw_adc));
}
/***************************** MQCalibration ****************************************
Input:   mq_pin - analog channel
Output:  Ro of the sensor
```

```
Remarks: This function assumes that the sensor is in clean air. It use MQResistanceCalculation to
calculates the sensor resistance in clean air and then divides it with RO_CLEAN_AIR_FACTOR.
RO_CLEAN_AIR_FACTOR is about 10, which differs slightly between different sensors.
************************************************************************************/
float MQCalibration(int mq_pin){
  int i;
  float val=0;
  for (i=0;i<CALIBARAION_SAMPLE_TIMES;i++) {         //take multiple samples
    val += MQResistanceCalculation(analogRead(mq_pin));
    delay(CALIBRATION_SAMPLE_INTERVAL);
  }
  val = val/CALIBARAION_SAMPLE_TIMES;                 //calculate the average value
  val = val/RO_CLEAN_AIR_FACTOR;                      //divided by RO_CLEAN_AIR_FACTOR yields the Ro
                                                      //according to the chart in the datasheet
  return val;
}
/*****************************  MQRead *********************************************
Input:   mq_pin - analog channel
Output:  Rs of the sensor
Remarks: This function use MQResistanceCalculation to caculate the sensor resistenc (Rs). The Rs changes
as the sensor is in the different consentration of the target gas. The sample times and the time interval
between samples could be configured by changing the definition of the macros.
************************************************************************************/
float MQRead(int mq_pin){
  int i;
  float rs=0;
  for (i=0;i<READ_SAMPLE_TIMES;i++) {
    rs += MQResistanceCalculation(analogRead(mq_pin));
    delay(READ_SAMPLE_INTERVAL);
  }
  rs = rs/READ_SAMPLE_TIMES;
  return rs;
}
/*****************************  MQGetGasPercentage **********************************
Input:   rs_ro_ratio - Rs divided by Ro, gas_id - target gas type
Output:  ppm of the target gas
Remarks: This function passes different curves to the MQGetPercentage function which calculates the
ppm (parts per million) of the target gas.
************************************************************************************/
int MQGetGasPercentage(float rs_ro_ratio, int gas_id){
  if ( gas_id == GAS_H2) {
     return MQGetPercentage(rs_ro_ratio,H2Curve);
  }
  return 0;
}
/*****************************  MQGetPercentage **********************************
Input:   rs_ro_ratio - Rs divided by Ro, pcurve-pointer to the curve of the target gas
Output:  ppm of the target gas
Remarks: By using the slope and a point of the line. The x(logarithmic value of ppm) of the line could be
derived if y(rs_ro_ratio) is provided. As it is a logarithmic coordinate, power of 10 is used to convert the
result to non-logarithmic value.
************************************************************************************/
int  MQGetPercentage(float rs_ro_ratio, float *pcurve){
  return (pow(10,( ((log(rs_ro_ratio)-pcurve[1])/pcurve[2]) + pcurve[0])));
}
```

References

[S1] D. T. Duong, C. Wang, E. Antono, M. F. Toney, A. Salleo, *Organic Electronics* **2013**, 14, 1330-1336.

[S2] P. J. Stephens, F. J. Devlin, C. F. Chabalowski, M. J. Frisch, *J. Phys. Chem.* **1994**, 98, 45, 11623–11627

[S3] J. R. Thomas, B. J. DeLeeuw, G. Vacek, T. D. Crawford, Y. Yamaguchi, H. F. Schaefer, *J. Chem. Phys.* **1993**, 99, 403–416

[S4] A. V. Marenich, C. J. Cramer, D. G. Truhlar, *J. Chem. Theory Comput.* **2013**, 9, 1, 609–620

[S5] SCM, SM12: Solvation Model 12, accessed 16 May 2024, <https://www.scm.com/doc/ADF/Input/SM12.html?highlight=SM12#ref1>

[S6] Sandbox Electronics, MQ-8 Hydrogen/H2 Sensor Module, accessed 14 September 2023, <https://sandboxelectronics.com/?p=196>
